# DFT Insights into the Role of Relative Positions of Fe and N Dopants on the Structure and Properties of TiO_2_

**DOI:** 10.3390/ma11020313

**Published:** 2018-02-22

**Authors:** Sahar Ramin Gul, Matiullah Khan, Zeng Yi, Bo Wu

**Affiliations:** 1 State Key Lab of High Performance Ceramics and Superfine Microstructure, Shanghai Institute of Ceramics, Chinese Academy of Sciences, Shanghai 200050, China; srgmk14@gmail.com (S.R.G.); matiullahustb@gmail.com (M.K.); 2 Multiscale Computational Materials Facility, College of Materials Science and Engineering, Fuzhou University, Fuzhou 350100, China; 3 Department of Physics, Kohat University of Science and Technology (KUST), Kohat 26000, Pakistan

**Keywords:** density functional theory, Fe, N-TiO_2_, optical properties

## Abstract

The location and nature of the doped elements strongly affect the structural, electronic and optical properties of TiO_2_. To tailor the band structure and modify the photoelectrochemical properties of TiO_2_, a pair of dopants is selected. Fe and N atoms are inserted in the TiO_2_ network at substitutional and interstitial sites with different relative distances. The main objective behind the different locations and sites of the doped elements is to banish the isolated unoccupied states from the forbidden region that normally annihilates the photogenerated carriers. Fe at the Ti site and N at the O site doped in the TiO_2_ network separated at a distance of 7.805 Å provided a suitable configuration of dopant atoms in terms of geometry and band structure. Moreover, the optical properties showed a notable shift to the visible regime. Individual dopants either introduced isolated unoccupied states in the band gap or disturbed the fermi level and structural properties. Furthermore, the other co-doped configurations showed no remarkable band shift, as well as exhibiting a suitable band structure. Resultantly, comparing the band structure and optical properties, it is argued that Fe (at Ti) and N (at O) doped at a distance of 7.805 Å would strongly improve the photoelectrochemical properties of TiO_2_.

## 1. Introduction

The location and nature of dopant elements plays a crucial role in tailoring the band structure of TiO_2_. Nitrogen has been widely used as a dopant element since the report by Sato [1], and is considered to be a promising source for improving the application spectrum of TiO_2_ [2,3,4,5]. The location of the N dopant in the TiO_2_ network strongly influences the band structure. Substitutional N doping at Ti sites introduces N 2p states at 0.14 eV above the top of the valence band, and inserting N at the interstitial sites creates N 2p at 0.73 eV above the valence band maximum [6]. Moreover, replacing O^2−^ with N^3−^ leads to charge imbalance in the TiO_2_ network.

Wide functionalities of the TiO_2_ could be achieved by reducing the band gap and improving the separation between the photoexcited carriers. Different elements from the periodic table are doped in TiO_2_ to improve its photocatalytic activity [7,8,9,10,11,12]. Doping two elements simultaneously improves the photon absorption (visible light range) and the photocatalytic activity compared to mono-doping [13,14].

Doping transition metals into TiO_2_ has been widely reported in the literature, and has been found to improve the optical response of TiO_2_. However, in some cases, the metal ions create states below the conduction band minimum, which helps in eliminating the photoexcited carriers [15]. Transition metal doping in N-doped TiO_2_ is expected to improve the separation between the photoexcited electrons and holes [16]. Fe and N co-doping in the TiO_2_ lattice has been studied experimentally [17] as well as theoretically [18]. However, there is a lack of theoretical understanding about the effect of dopant location on the band structure and optical properties. Moreover, it is also necessary to address the charge compensation due to doping effect in detail. 

Addressing the configurations of dopant in the TiO_2_ network, this manuscript reports the effect of dopant location on the optical properties of co-doping modeled system. Along with the mono-doped systems, three different Fe, N co-doped TiO_2_ systems are modeled. Geometrical structure is linked with the electronic band structure and optical properties in order to elucidate the Fe, N co-doped TiO_2_ systems. 

## 2. Materials and Methods

With the Cambridge Serial Total Energy Package (CASTEP) code [19], generalized gradient approximation (GGA)-based calculations are performed, where the electron wave functions are expanded in plane waves. Interactions between the ions and electrons are modeled by ultrasoft pseudopotential [20]. Moreover, the atomic positions are optimized by Broyden-Fletcher-Goldfarb-Shanno (BFGS) energy minimization algorithm. In order to relax the structure of the modeled systems, the constraints in the form of maximum force, maximum stress and maximum displacement were 0.01 eV/Å, 0.1 GPa, and 0.01 Å, respectively [21]. 

**Figure 1:**
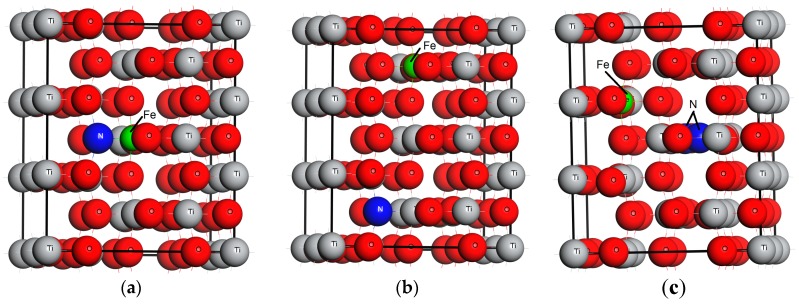
Relative positions of Fe and N dopant in the structure of TiO_2_, (**a**) Model A, (**b**) Model B, and (**c**) Model C.

Anatase TiO_2_ model with 3 × 2 × 1 replication, have 72 atoms, having 24 Ti and 48 O atoms. Location of dopant strongly affects the electronic band structure and optical properties. Three configurations for co-doping Fe and N in the TiO_2_ network were modeled, based on the relative positions of dopants and doping sites. Model A, with Fe and N atoms doped at the Ti and N sites, respectively are 1.739 Å apart from each other. Replacing single Ti with Fe and single O with N atoms, located at a distance of 7.805 Å is represented as Model B. Inserting two N atoms at the interstitial sites separated by a distance of 2.21 Å, along with Fe at the Ti site in 3 × 2 × 1 supercell is named Model C. The Fe, N-TiO_2_ modeled systems are displayed in Figure 1. Monodoping of Fe and N in the TiO_2_ network is modeled by replacing the lattice Ti and O atoms, respectively. Substitutional Fe doped at the Ti site in the anatase TiO_2_ supercell is represented by FeT. Replacing the lattice O atom with an N atom provides a N-doped TiO_2_ model symbolized as NT. As a reference model, the pure anatase TiO_2_ denoted by PT is also simulated. 

## 3. Results and Discussion

### 3.1. Geometrical Structure of the Modeled Systems

Optimized TiO_2_ has lattice parameters of: a = 3.80727 Å, b = 3.80538 Å and c = 9.68152 Å. The calculated data agree with the theoretical findings [22], but these are overestimated when compared with the experimental results [23]. Optimized bond lengths are summarized in Table 1. The O-Ti (1.9622 Å) bond length showed decreasing trend due to doping except the Model B which depicted small deviation from the corresponding value of pure TiO_2_. As can be seen from Table 1, the O-O bond lengths of the doped modeled systems are elongated in reference to bare TiO_2_. The cause might be the difference of ionic radii of Fe, N and Ti [24]. It is quite difficult to justify the relative distortion from the bond lengths because new bonds are created in the doped systems. However, comparing the bond lengths of model B with the other modeled systems and pure TiO_2_, it is expected that this model might provide minimum structure distortion. 

### 3.2. Band Structure and Partial Density of States of the Modeled Systems

**Figure 2:**
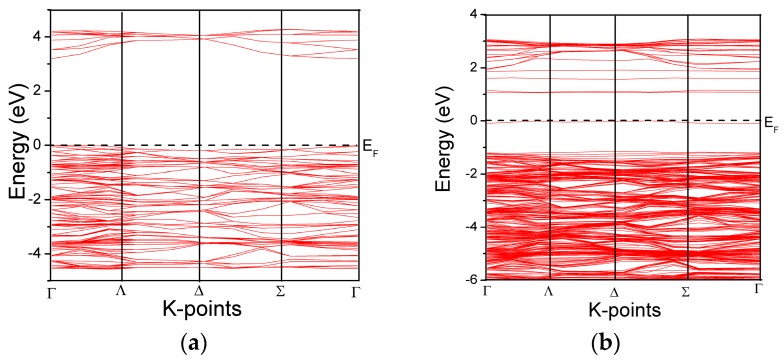

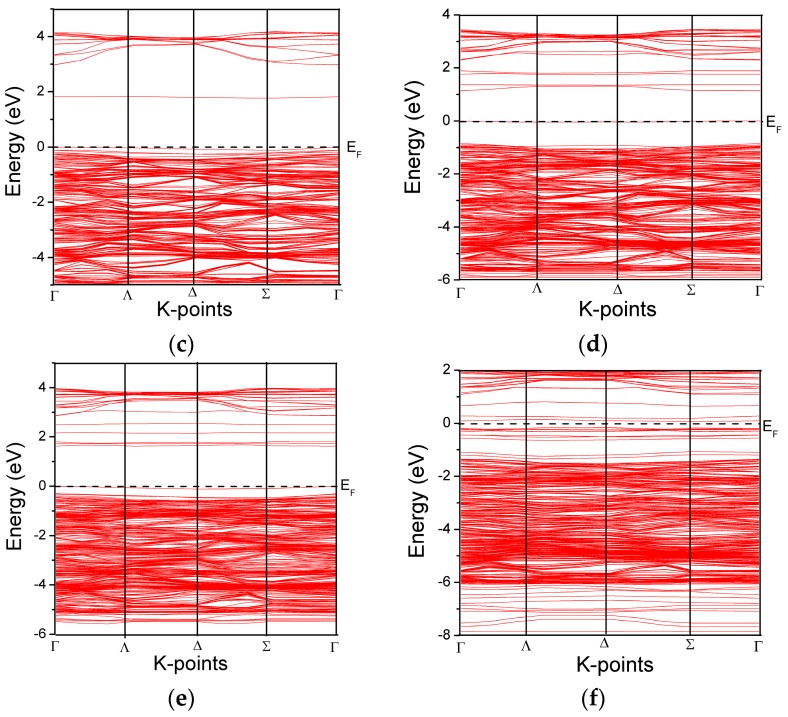
Electronic band structure: (**a**) PT, (**b**) FeT, (**c**) NT, (**d**) Model A, (**e**) Model B, and (**f**) Model C.

The band structures of the modeled doped systems are plotted in Figure 2. Calculated using conventional density functional theory (DFT), the band gaps are underestimated. However, it is updated using scissor approximation [25] in order to make a comparison with the experimental data [26]. The calculated band gaps of the different modeled systems are summarized in Table 2. Pure semiconducting state is evident from the band structure of bare TiO_2_, as the Fermi level is present just above the valence band maximum (VBM). As seen from Figure 2b, substitutional Fe doping at Ti sites creates isolated states below the conduction band minimum (CMB), and the fermi level is shifted up in the band gap. Strong absorption is expected from this modeled system; however, it might not contribute well to the photoactivity due to the isolated nature of the states below the conduction band minimum. N doping introduces unoccupied states in the band gap, which is consistent with the literature [27,28]. The co-doped model A band structure is not much modified compared to FeT, and the fermi level is present at 0.85 eV above the top of valence band. It is interesting to note that model B lowers the position of the fermi level in the band gap (0.3 eV above VBM). Moreover, as depicted in Figure 2e, the states below the CBM are mixed with the conduction band, which might improve the photoactivity and photon absorption in the visible regime. Table 2 shows that the reduction in the band gap of model B is also notable. Model C displays a metallic character because the CBM and VBM move closer to one another, and many states are introduced in the band gap. 

**Figure 3:**
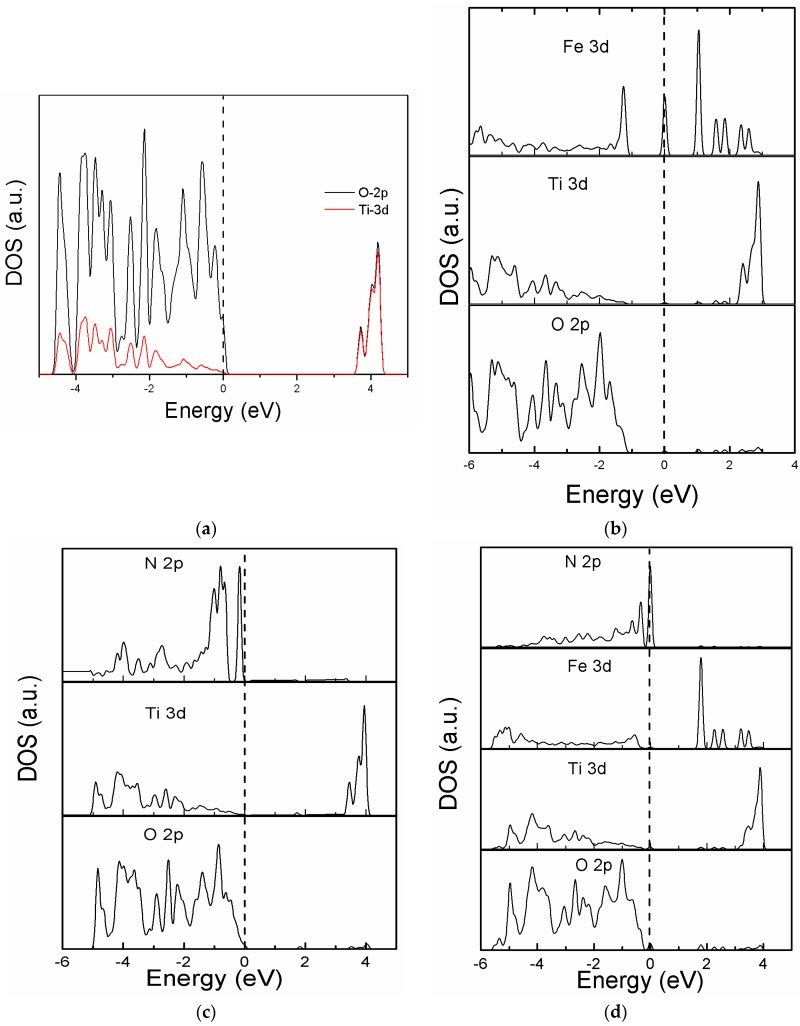
Partial density of states (PDOS) of: (**a**) PT, (**b**) FeT, (**c**) NT, and (**d**) Model B.

The partial density of states (PDOS) of the modeled systems is shown in Figure 3. As is evident from Figure 3a, the valence band of TiO_2_ comes from O 2p states, and the conduction band is composed of Ti 3d states. Fe doping modifies the band structure, and the Fe 3d states are introduced in the forbidden region, which is in agreement with the reported data [29]. Without altering the position of the fermi level, the N 2p states reduces the band gap of TiO_2_. One should note that Fe 3d and N 2p states are introduced simultaneously into the band structure of Model B. The role of the Fe 3d states is very interesting, because they reduce the band gap by mixing with the Ti 3d states and push the CBM to low energy values. Moreover, the 2p states of nitrogen are coupled with the O 2p states, leading to a reduction in the band gap. It is interesting to note that the N 2p states in Figure 3d are occupied, and they would not contribute to the elimination of electron-hole pairs. Therefore, the role of N 2p states in Model B is different from its role in NT, because in the former case, it is occupied, while in the latter case, it is unoccupied. Electron transfer from the fully occupied states to CBM reduces the transition energy of photons, leading to improved separation between the photoexcited carriers. The PDOS analysis indicates that the intrinsic band structure of TiO_2_ can be tailored by selecting the nature and proper location of the dopants. 

**Figure 4:**
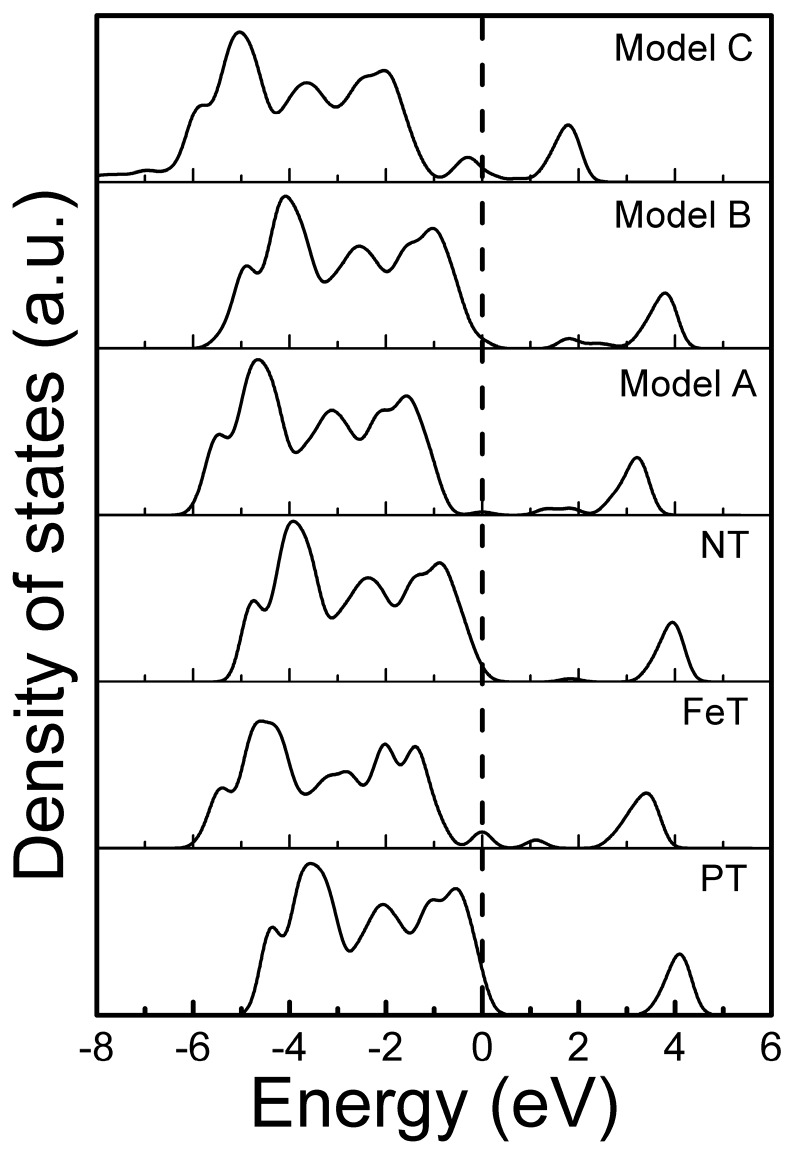
Comparison of the total density of states of simulated modeled systems, in reference to bare TiO_2_.

Total density of states of the modeled systems are compared and depicted in Figure 4. Except NT and Model B, none of doped modeled systems retains the position of the fermi level at VBM. In the case of NT, because the isolated states introduced in the band gap are unoccupied, it may therefore eliminate the photogenerated carriers. The band structure of model B is considered suitable for utilizing this system in the degradation of inorganic materials under visible light irradiations.

### 3.3. Photo-Response of the Modeled Systems

Photons (suitable frequency) interact with the electrons of TiO_2_, shifting them from the valence to the conduction band. The transitions of electrons between the occupied and unoccupied states produce spectra and optical properties originating from such transitions. CASTEP calculates the optical properties from the complex dielectric function consisting of real part $\varepsilon_{1}\left(\omega \right)$ and imaginary part $\varepsilon_{2}\left(\omega \right)$. The real and imaginary parts are used to calculate the absorption coefficient using Equation (1) [30,31].

(1) $$\alpha \left(\omega \right)=\sqrt{2}\omega {\left[\sqrt{\varepsilon_{1}^{2}\left(\omega \right)+\varepsilon_{2}^{2}\left(\omega \right)}-\varepsilon_{1}\left(\omega \right)\right]}^{\frac{1}{2}}$$

**Figure 5:**
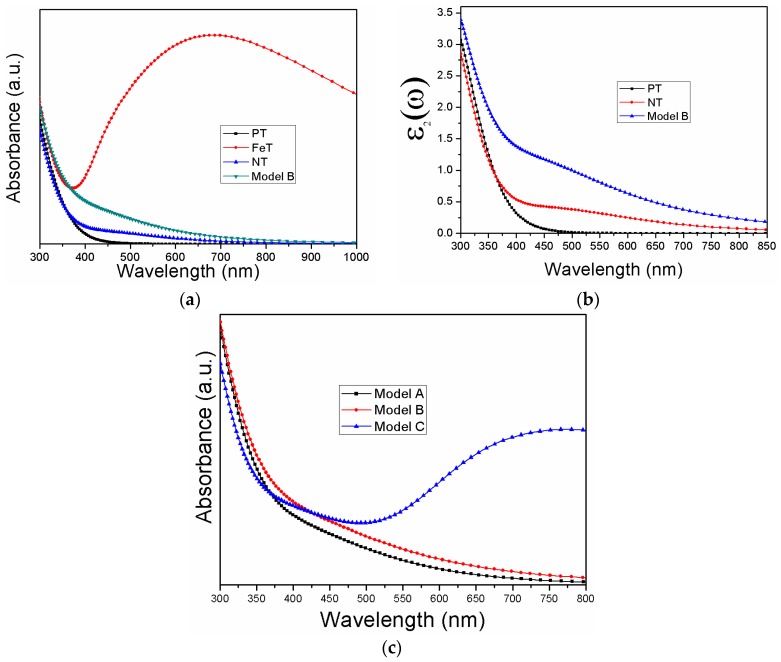
Photo-response of the simulated systems (**a**) absorption, (**b**) imaginary part of the dielectric function, and (**c**) absorption of the co-doped systems.

The photo-response of the simulated models is depicted in Figure 5. As can be seen from Figure 5a, the bare TiO_2_ is only sensitive to ultra violet (UV) light, having no absorption in the visible regime. This spectrum is attributed to the transition of electrons between O 2p and Ti 3d states. Dopant elements introduce states in the band gap that affect the absorption curve of TiO_2_. Both FeT and NT are sensitized to visible as well as UV light. In the case of FeT, the visible photons absorption originates from the shifting of electrons from O 2p to Ti 3d through Fe 3d states. Step-wise transition occurs in NT. Initially, the electrons are excited to N 2p states from O 2p states, and further move to Ti 3d states, completing the absorption curve. It is notable that the absorption of the modeled co-doping systems is prominent compared to the mono-doped systems. Both N 2p and Ti 3d states contribute to the shifting of electrons between O 2p and Ti 3d states. The results of the imaginary part of the dielectric function (Figure 5b) are in accordance with the absorption coefficient spectra. One should note from Figure 5c that model C provided a hump of absorption that might be due to the interstitial positions of the doped nitrogen atoms. This absorption might not contribute well to the photoactivity because the photogenerated carriers would soon be ready for elimination. Comparing the absorption threshold of the co-doping systems, model B is expected to have the highest efficiency among the modeled systems in photoelectrochemical applications. Along with its stable structure and clear band gap, it exhibits visible light absorption, thus utilizing the major spectrum of solar light.

## 4. Conclusions

With density functional theory-based calculations, the structure and properties of the mono-doped and co-doped models of Fe and N were calculated. Mono-doping either introduced isolated unoccupied states in the band gap or disturbed the fermi level. Fe doping shifted the fermi level from the top of the valence band to the middle of the forbidden region. Moreover, some isolated states appeared below the conduction band minimum. Along with some isolated states, N doping at O sites reduced the band gap of TiO_2_ form 3.20 eV to 2.867 eV. Fe and N doped adjacent to each other (1.739 Å apart) at Ti and O sites, respectively, shifted the fermi level up in the band gap, along with creating isolated states below the conduction band minimum. Without creating considerable changes in the fermi level and reducing the band gap of TiO_2_ to 1.798 eV, the substitutional Fe (at Ti sites) and N (at N sites) separated by 7.805 Å provided a suitable band structure and improved the optical response in the visible regime. This model is considered the best among the simulated models, and it can reasonably explain the experimental findings.

## Figures and Tables

**Table 1:** Optimized bond lengths (Å) of Fe and/or N doping modeled systems.

Systems	O-Ti	O-O	O-Fe	Fe-Ti	O-N	N-Ti	Fe-N	Ti-Ti	N-N
PT	1.9622	2.6346	-	-	-	-	-	-	-
FeT	1.9488	2.6990	1.8900	2.9945	-	-	-	-	-
NT	1.9473	2.6945	-	-	2.6675	2.0390	-	-	-
Model A	1.9479	2.6766	1.9289	2.9684	2.8328	2.0059	2.9684	-	-
Model B	1.9520	2.7025	1.8739	-	2.8018	1.8951	-	2.9780	-
Model C	1.9479	2.7035	1.8940	2.8534	2.2241	2.7725	-	2.9377	2.2207

**Table 2:** Band gap of Fe- and/or N-doped modeled systems.

	PT	FeT	NT	Model A	Model B	Model C
**Band gap (eV)**	3.20	2.061	2.867	1.851	1.798	0.527
